# Modelling the Adhesion and Biofilm Formation Boundary of *Listeria monocytogenes* ST9

**DOI:** 10.3390/foods11131940

**Published:** 2022-06-29

**Authors:** Lili Hu, Qingli Dong, Zhuosi Li, Yue Ma, Muhammad Zohaib Aslam, Yangtai Liu

**Affiliations:** School of Health Science and Engineering, University of Shanghai for Science and Technology, Shanghai 200093, China; usst_hll@163.com (L.H.); qdong@usst.edu.cn (Q.D.); lizhuosi@usst.edu.cn (Z.L.); yuema@usst.edu.cn (Y.M.); zohaib.aslam.000@gmail.com (M.Z.A.)

**Keywords:** *Listeria monocytogenes*, adhesion, biofilm formation, boundary model

## Abstract

*Listeria monocytogenes* is a major foodborne pathogen that can adhere to or form a biofilm on food contact surfaces, depending on the environmental conditions. The purpose of this work is to determine the adhesion and biofilm formation boundaries for *L. monocytogenes* ST9 under the combination environments of temperature (5, 15, and 25 °C), NaCl concentration (0%, 3%, 6%, and 9% (*w/v*)) and pH (5.0, 6.0, 7.0, and 8.0). The probability models of adhesion and biofilm formation were built using the logistic regression. For adhesion, only the terms of linear T and NaCl are significant for *L. monocytogenes* ST9 (*p* < 0.05), whereas the terms of linear T, NaCl, and pH, and the interaction between T and pH were significant for biofilm formation (*p* < 0.05). By analyzing contour maps and their surface plots for two different states, we discovered that high temperature promoted adhesion and biofilm formation, whereas excessive NaCl concentration inhibited both of them. With a stringent threshold of 0.1667, the accuracy rate for identifying both adhesion/no-adhesion and biofilm formation/no-biofilm formation events were 0.929, indicating that the probability models are reasonably accurate in predicting the adhesion and biofilm formation boundary of *L. monocytogenes* ST9. The boundary model may provide a useful way for determining and further controlling *L. monocytogenes* adhesion and biofilm formation in various food processing environments.

## 1. Introduction

*Listeria monocytogenes* is a concerning pathogen widely distributed in foods and associated environments, resulting in severe illnesses and deaths [1,2]. Between 2011 and 2017, 136 relevant outbreaks were reported and 562 patients were diagnosed with listeriosis in mainland China [3]. The meat production and consumption chain was widely regarded as one of the primary routes for the spread of *L. monocytogenes* [4,5]. According to the Chinese national microbiological food safety surveillance network, between 2008 and 2016, the prevalence of *L. monocytogenes* in poultry-meat products was up to 8.91%, which was the highest food among 14 categories [6]. Several investigations revealed that the predominant sequence types (STs) of *L. monocytogenes* in meats or their processing plants is ST9 [6,7,8,9]. *L. monocytogenes* ST9 may persist in the form of planktonic or biofilm in foods or on food contact surfaces, constituting a continuous source of contamination during meat production.

Biofilm is an aggregation of microorganisms attached to a surface and embedded in a matrix of extracellular polymeric substances (EPS), which is a common pattern of microbial persistence [10,11]. Once the adhered bacterial population develops into a matured biofilm, it can be more resistant to various stresses and more difficult to eradicate, compared with the planktonic form [12,13]. In particular, biofilms have been observed to be 10 to 1000 times more resistant to various antibiotics [11,14]. A biofilm lifecycle is divided into four stages: adhesion; microcolony formation; maturation; and dispersion. Such a dynamic process is affected by various factors, including strain characteristics, temperature, pH, nutrition, osmotic pressure, etc. [15]. Previous studies have discovered that *L. monocytogenes* biofilm shows a variety of structures: mono-layers of adhered cells; flat unstructured multilayers; honeycomb structures; or clusters with EPS [16]. The components of EPS, containing proteins, extracellular polysaccharides, DNA, and teichoic acid, can provide nutrients and allow cell-to-cell communication for the bacteria [17]. Due to its strong antibiotic resistance, virulence, and persistence, biofilm has become a significant threat to food safety. It is meaningful to build a model to describe how the environmental factors affect bacterial adhesion and biofilm formation.

Recently, the growth/no growth interfaces were extensively modeled for planktonic spoilages and bacteria as a function of food-related environments, such as temperature, pH, and NaCl concentration [18,19]. Such boundary models can estimate the growth limitations of microorganisms under specific environmental conditions, which might provide a realistic evaluation of food safety. Several models with diverse factors of *L. monocytogenes* were developed in a liquid laboratory broth, or even in a real food matrix [20,21]. For instance, Schvartzman et al. demonstrated that the growth/no growth interfaces in broth, milk, and cheese were different, and that the cheesemaking conditions could promote a lower growth of *L. monocytogenes* than the broth and milk [22]. These studies provided useful information for microbial kinetic analysis and further quantitative risk assessment in foods. However, there are limited studies on developing the biofilm formation boundary for *L. monocytogenes*, especially considering the environmental factors in the meat chain [23,24].

Therefore, our research intends to determine the adhesion and biofilm formation boundary of *L. monocytogenes* ST9 under the conditions of temperature, pH, and NaCl. The observations and established models are expected to contribute to a better understanding of the persistent contamination of *L. monocytogenes* during meat processing.

## 2. Materials and Methods

### 2.1. Preparation of L. monocytogenes Suspension

A strain of *L. monocytogenes* ST9 (lineage II), isolated from Chinese meat products, was employed in this study. This strain was formerly reported by Tian et al. (Strain no. 13) and showed a relative short lag phase and strong biofilm-forming ability, compared to others [25]. The strain was frozen in glycerol stock culture and inoculated into Tryptone Soy Broth with 0.6% Yeast Extract (TSB-YE; Hopebio Technology Co. Ltd., Qingdao, China). The working stocks were stored at 4 °C on Tryptone Soy Agar with 0.6% Yeast Extract (TSA-YE; Hopebio) for four weeks. Prior to each experiment, a single colony was inoculated into the TSB-YE at 37 °C overnight to yield late stationary phase cells. The cells were harvested by centrifugation at 8000× *g* for 10 min at 4 °C (Thermo Fisher Scientific Co. Ltd., Shanghai, China). The harvested cells were washed three times and re-suspended with 1 mL of 0.85% sterile saline solution. Each suspension was diluted with 0.85% saline solution to a concentration of 10^6^ CFU/mL for the following tests.

### 2.2. Incubation of L. monocytogenes

To simulate the major environments in meat processing plants, three primary factors of pH values (5.0, 6.0, 7.0, and 8.0), NaCl concentration (0%, 3%, 6%, and 9% (*w/v*)), and temperature (5, 15, and 25 °C) were used as the control factors. The above factors were combined into 48 conditions, following the full factorial design for obtaining the adhesion and biofilm formation characteristics of *L. monocytogenes*. The observed data under 48 conditions were randomly assigned in a 7:3 ratio into a modeling group (containing 34 conditions) and a validation group (containing 14 conditions). The salt concentration and pH value of sterile TSB-YE was adjusted by adding NaCl and using HCl or NaOH (2 mol/L), respectively [26]. Then, each 180 μL of the developed medium was transferred into each well of 96-well polystyrene microplates, with 20 μL prepared suspension [27,28]. The microplates were incubated at 5, 15, and 25 °C for 48 h, prior to biofilm testing.

### 2.3. Enumeration and Evaluation of L. monocytogenes

Following the incubation, the TSB-YE from each well was discarded and rinsed three times with 0.85% saline solution to remove the no-adhered cells. Then, each well was thoroughly scraped with sterile cotton swabs. The swabs were immersed in 5 mL of saline solution and vortexed for about 2 min with beads [28]. Subsequently, the decimal dilutions were prepared and plated, and the plates were incubated at 37 °C for 24 h. The results were reported as log_10_ CFU/mL. The *L. monocytogenes* population was considered as adhesion when the counts of each well were 3.00 to 5.00 log_10_ CFU/mL, otherwise it was considered as biofilm formation when greater than 5.00 log_10_ CFU/mL [29,30]. Each combination of pH, temperature, and NaCl was performed in six replicates to calculate the probability of adhesion or biofilm formation under a specific condition.

### 2.4. Adhesion and Biofilm Formation Boundaries Modelling of L. monocytogenes

A logistic regression model, to describe the boundary of *L. monocytogenes* adhesion or biofilm formation, was applied and listed as follows:

(1) $$\begin{array}{ll} \mathrm{Logit}\left(P\right) & =\ln\left(\frac{P}{1-P}\right)\\ & =a_{0}+a_{1}\times T+a_{2}\times \mathrm{NaCl}+a_{3}\times \mathrm{pH}+a_{4}\times T\times \mathrm{NaCl}\\ & +a_{5}\times T\times \mathrm{pH}+a_{6}\times \mathrm{NaCl}\times \mathrm{pH}+a_{7}\times T^{2}+a_{8}\times {\mathrm{NaCl}}^{2}\\ & +a_{9}\times {\mathrm{pH}}^{2} \end{array}$$

where *P* is the probability of adhesion and biofilm formation; $a_{i}$ is the parameters to be estimated; pH is the pH value of the medium; T (°C) is temperature; and NaCl (% (*w/v*)) is the NaCl concentration, respectively. When the *L. monocytogenes* counts of 3.00 and 5.00 log_10_ CFU/mL, or more than 5.00 log_10_ CFU/mL, are found, *P* is assigned the value of 1 at a specific combination of three factors. Meanwhile, a value of 0 was assigned when the counts of *L. monocytogenes* were 0 to 3.00 log_10_ CFU/mL, which indicates the absence of adhesion or biofilm formation.

The Minitab 19.0 software (Minitab Inc., State College, PA, USA) was used to analyze the data. A stepwise selection procedure was used to determine the significant parameters (*p* < 0.05). The adhesion/no-adhesion or biofilm formation/no-biofilm formation interfaces were constructed when the logistic regression models were fitted. The performance of the obtained models was evaluated with the adjusted *R^2^* and the Bayesian information criterion (BIC). The equations were listed as follows, respectively [31]:

(2) $$\mathrm{Adjusted}\;R^{2}=1-\left(\frac{n-1}{n-p}\right)\left(\frac{\mathrm{SSE}}{\mathrm{SST}}\right)$$

(3) $$\mathrm{BIC}=n\ln\left(\frac{\mathrm{SSE}}{n}\right)+p\;\ln\left(n\right)$$

where *n* is the number of observed values; *p* is the number of parameters of the model; SSE is the sum of squares of errors; SST is the total sum of squares.

### 2.5. Experimental Validation of the Probability Models

According to the assignment rule (7:3 ratio), 14 randomly selected environmental combinations were performed for model validation. The adhesion or biofilm formation was determined to occur when one positive result was observed in the six replications. Thus, the threshold of adhesion or biofilm formation was set as 0.1667 (=1/6) [32]. Then, the predicted probabilities were compared to the observations using the indicators of accuracy rate, precision rate, and root-mean-square error (RMSE). The equations for the above indicators were listed as follows, respectively:

(4) $$\mathrm{Accuracy}\;\mathrm{rate}=\frac{\mathrm{TPR}+\mathrm{TNR}}{\mathrm{TPR}+\mathrm{TNR}+\mathrm{FPR}+\mathrm{FNR}}$$

(5) $$\mathrm{Precision}\;\mathrm{rate}=\frac{\mathrm{TPR}}{\mathrm{TPR}+\mathrm{FPR}}$$

where TPR is the true positive rate; TNR is the true negative rate; FPR is the false positive rate; and FNR is the false negative rate, respectively:

(6) $$\mathrm{RMSE}=\sqrt{\frac{1}{n}\overset{n}{\underset{i=1}{\sum }}{\left(P_{o}-P_{p}\right)}^{2}}$$

where *n* is the number of observed values; *P_o_* is the observed probability of adhesion or biofilm formation; and *P_p_* is the predicted probability of adhesion or biofilm formation, respectively.

## 3. Results and Discussion

### 3.1. Development of Adhesion and Biofilm Formation Boundary Models

Among the 204 combinations of the conditions assayed, adhesion was observed in 52.5% (=107/204), biofilm formation was observed in 31.9% (=65/204). The terms that were not significant (*p* ≥ 0.05) were excluded from the logistic regression models, using a stepwise selection procedure. For adhesion, only the terms of linear T and *NaCl* are significant for *L. monocytogenes* ST9 strain (*p* < 0.05). The adhesion boundary model was listed as follows:

(7) $$\mathrm{Logit}\left(P_{\mathrm{adhesion}}\right)=-1.418+0.366\times T-0.721\times \mathrm{NaCl}$$

The estimated parameters and the standard error are shown in Table 1. According to the Wald test, the χ^2^ of *T* and NaCl were 51.650 and 28.490, indicating that the temperature had the most essential effect for the adhesion of *L. monocytogenes* ST9, followed by the NaCl concentration. Within the investigated pH values, the influence of pH on adhesion appeared to be insignificant. Adhesion is an important early stage of the biofilm formation process, and is driven by cell motility or Brownian motion. The sessile cells can initiate the next stage, microcolony formation, by utilizing pili, flagella, or exopolysaccharides’ production [33].

As regards the biofilm formation, the terms of linear T, NaCl, and pH, the interaction between T and pH was significant (*p* < 0.05). The biofilm formation boundary model was listed as follows, and the estimated parameters were shown in Table 2:

(8) $$\mathrm{Logit}\left(P_{\mathrm{biofilm}}\right)=8.260-0.795T-0.754\times \mathrm{NaCl}-2.860\times \mathrm{pH}\;+0.235\times T\times \mathrm{pH}$$

The χ^2^ of T, pH, NaCl and the interaction between T and pH were 2.570, 4.090, 23.050, and 7.440, based on the Wald test. The values of χ^2^ demonstrated that the NaCl concentration was the most significant factor, followed by the interaction between T and pH. Although the regression coefficient of T was not significant (*p* > 0.05), the regression model was acceptable, according to the BIC.

**Figure 1:**
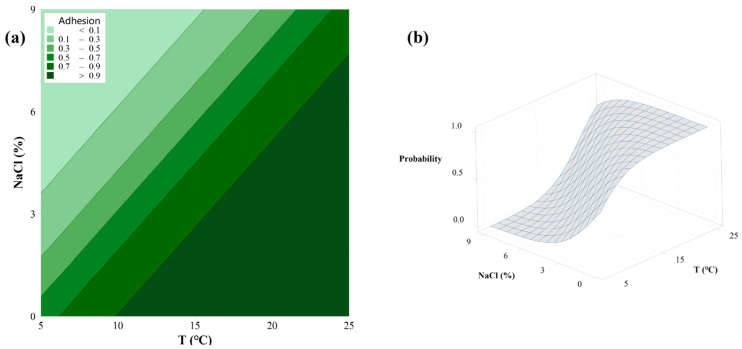
Contour map (**a**) and surface plot (**b**) of adhesion probability by L. monocytogenes ST9, as affected by T and NaCl.

The probability of adhesion of the *L. monocytogenes* ST9, as affected by T and NaCl, is shown in Figure 1. From the contour map, the darker green in the area represented a higher adhesion probability. The surface plot can directly reflect the trend of adhesion probability with three environmental factors. High temperatures could promote adhesion, whereas excessive NaCl concentration inhibited it. For instance, the probability of adhesion was less than 0.1 when the NaCl concentration reached up to 6% at 5 °C. While at the same NaCl concentration, it could be observed that the probability increased to 0.9 at 25 °C. Despite the fact that NaCl concentration was 6% higher, the probability of adhesion was higher than 0.9. Previous studies have proved that high temperatures could increase the metabolism of *L. monocytogenes* cells, which was directly associated with an increase in the cell division rates [34]. Additionally, the temperature can influence the cell surface structures, including the flagella, curli, surface proteins, and cell hydrophobicity, all of which are relevant for initial adhesion during the biofilm formation process [33]. The effect of salt on bacteria is responsible for increasing osmotic pressure, which requires more metabolic energy to prevent water loss via the accumulation of osmoprotectants [35]. Iliadis et al. also found that increasing the NaCl concentration could suppress the growth of *Salmonella* Enteritidis and *S*. Typhimurium sessile cells in low nutrient food-related conditions [26]. Additionally, it has been demonstrated that extremely high salt concentrations repressed flagella expression; thus, reducing the adhesion capability of *L. monocytogenes* to stainless steel and polystyrene [36].

**Figure 2:**
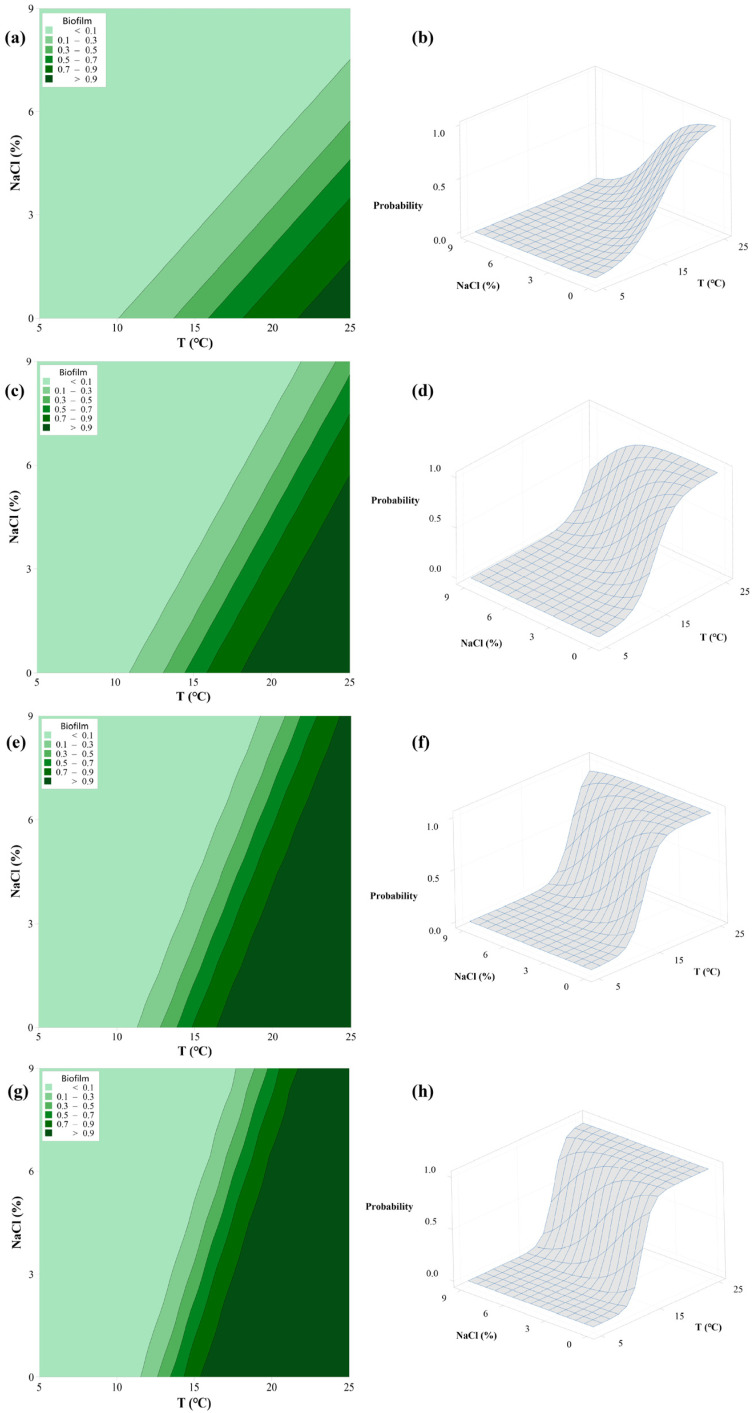
Contour maps and surface plots of biofilm formation probability by *L. monocytogenes* ST9 as affected by T and NaCl at pH 5.0 (**a**,**b**); pH 6.0 (**c**,**d**); pH 7.0 (**e**,**f**); and pH 8.0 (**g**,**h**).

Figure 2 illustrates the probability of biofilm formation of *L. monocytogenes* ST9, as affected by T and NaCl at different pH levels. Compared with adhesion, the effect of temperature and NaCl concentration exhibited a similar effect on the biofilm formation, in which a higher temperature and lower NaCl concentration could facilitate the formation of the biofilm. Bezek et al. found that the biofouling of *L. monocytogenes* grown at 37 °C was higher than that at 22 °C, regardless of the glucose concentration and abiotic surface materials [37]. These results demonstrated that the temperature is a critical factor for biofilm formation. Biofilm formation is different from adhesion, so the probability significantly increased as the pH level rose at a constant temperature and NaCl concentration. For instance, when the temperature was 25 °C and the NaCl concentration was less than 3%, the biofilm formation probability was greater than 0.9 at pH 5.0. While the same probability could be reached when the temperature exceeded 15 °C, at pH 7.0. At pH 8.0, the boundary of the biofilm formation was slightly wider than at pH 7.0. This indicated that the weakly alkaline environments could enhance biofilm formation, compared with neutral environments. Nilsson et al. found that biofilm production significantly increased under alkaline (pH 8.5) culture conditions after 48 h incubation (*p* < 0.05) [38]. It could be reasonably hypothesized that biofilm is a bacterial survival strategy to slight stresses. Previous studies found that sub-lethal stress conditions can enhance the biofilm formation in specific *S. enterica* strains [39]. In this study, the probability of biofilm formation could reach 0.9 at 25 °C, with pH 5.0. These results suggested that *L. monocytogenes* may able to form biofilms on the equipment surfaces under acidic conditions. Due to the strong ability of *L. monocytogenes* to form biofilms, more stringent sanitization procedures should be considered in food processing environments. In addition, other foodborne pathogens, such as *Salmonella* and *Escherichia coli,* have been deployed in the biofilm boundary models, with other environmental factors. Moraes et al. developed models to predict the adhesion and biofilm formation for *Salmonella enterica* as a function of temperature, NaCl, and pH [30]. The models showed adequate performance in predicting the boundary of adhesion and biofilm formation. This study also emphasized the strain variability of boundary models among the five different serovars. Compared to the limitations for adhesion and biofilm formation, it appears that *L. monocytogenes* can adhere and form biofilm more readily than *Salmonella* at refrigerated temperatures. Perhaps the psychrophilic nature of *L. monocytogenes* permits it to adhere to, or form biofilm on, contact surfaces at low temperatures. Mendonça et al. established the boundary models for biofilm formation of *E. coli* O157:H7 by contact time and temperature, under different types of materials. The results showed that the material type, temperature, and contact time jointly affect the biofilm formation [40]. Additionally, Dimakopoulou et al. developed the probabilistic models to describe the biofilm formation boundary of *Salmonella* Newport under different combinations of pH and water activity at 37 °C [41]. However, the boundaries of *S*. Newport observed in this study are significantly wider in comparison with the growth boundaries of *Salmonella* reported in previous studies [42]. It demonstrated that bacteria can form biofilm under slightly stressful environments, which may not favor planktonic bacterial growth.

### 3.2. Model Validation

When probability models are used to determine whether *L. monocytogenes* may adhere to or form biofilm with different combinations of control factors, it is critical to use a criterion with high accuracy rate, low false positive rate, and low false negative rate. The models were used to judge adhesion/no-adhesion and biofilm formation/no-biofilm formation using the threshold (0.1667). The values predicted by the models developed for the prediction of adhesion and biofilm formation boundaries shows a high agreement with the values in the assays of the experimental validation (Table 3 and Table 4). The column of “*P_o_*” represents the observed adhesion or biofilm formation probability, *P_o_* ≥ 0.1667 indicates that the adhesion or biofilm formation of *L. monocytogenes* had occurred in the experiments.

In Table 3, ten tests were accurately classified as adhesion events, while test 40 and 42 were false positives. Besides, both test 36 and 45 were no-adhesion, from observation and prediction. According to the confusion matrix (Table 5), the overall accuracy rate is 0.929 in predicting adhesion. The TPR reaches to 1.000, the TNR is 0.857, and the precision rate is 0.875. Moreover, the RMSE between the observation value and the prediction value is 0.285. In Table 4, five tests had biofilm formation events and seven tests had no biofilm formation. Meanwhile, test 39 was false positive and test 47 was false negative. The accuracy rate of predicting biofilm formation is 0.929. The TPR and TNR values are 0.929, and the precision is 0.482. The RMSE is 0.291, indicating that biofilm formation was well predicted. In many cases, the disagreements between prediction and observation were false positives, which may be due to unknown factors related to adhesion and biofilm formation. Nevertheless, the results are relatively accurate (above 90%) to classify adhesion/no-adhesion and biofilm formation/no-biofilm formation. Then, the obtained models can be considered adequate for evaluating the adhesion and biofilm formation boundaries of *L. monocytogenes*. Furthermore, this also indirectly reflects the importance and effectiveness of hurdle technology in controlling biofilm formation of microorganisms in the meat processing.

## 4. Conclusions

This study evaluated the effect of temperature, pH, and NaCl concentration on the adhesion and biofilm formation interfaces of *L. monocytogenes* ST9. The logistic regression models showed adequate performance to predict the probability of the two statuses. The obtained results demonstrated that adhesion was affected by temperature and NaCl concentration, while biofilm formation was associated with temperature, pH, and NaCl concentration. The accuracy rate for classifying both the adhesion/no-adhesion and biofilm formation/no-biofilm formation events was 0.929, suggesting that the probability models are reasonably accurate in predicting the adhesion and biofilm formation boundaries of *L. monocytogenes* ST9. Overall, the developed boundary models can quantitatively describe the impact of temperature, NaCl concentration, and pH on the adhesion and biofilm formation of *L. monocytogenes*, thereby guiding the control measures to eliminate the sessile cells in meat processing and reducing the risk of foodborne listeriosis. Further research objectives should consider the influence of more environmental factors on the adhesion and biofilm formation boundary.

## Figures and Tables

**Table 1:** Logistic regression of combined effect of T, pH, and NaCl on adhesion probability of *L. monocytogenes* ST9.

	Estimated Coefficient	Standard Error	Wald χ^2^	*p*-Value
Constant	−1.418	0.721	52.500	0.000
T	0.366	0.051	51.650	0.000
NaCl	−0.721	0.135	28.490	0.000
Adjusted *R^2^*	0.640			
BIC	115.510			

**Table 2:** Logistic regression of combined effect of T, pH, and NaCl on biofilm formation probability of *L. monocytogenes* ST9.

	Estimated Coefficient	Standard Error	Wald χ^2^	*p*-Value
Constant	8.260	8.510	28.680	0.000
T	−0.795	0.496	2.570	0.109
NaCl	−0.754	0.157	23.050	0.000
pH	−2.860	1.410	4.090	0.043
T × pH	0.235	0.086	7.440	0.001
Adjusted *R^2^*	0.679			
BIC	104.450			

**Table 3:** Validation of logistic regression model and classification of adhesion and no-adhesion according to threshold (0.1667).

ID	T (°C)	NaCl (%)	pH	*P_o_* ^1^	*P_p_* ^2^	*P_o_* ≥ 0.1667 ^3^	*P_p_* ≥ 0.1667 ^4^	False Positive	False Negative
35	25	3	6.0	1.0000	0.9962	Y	Y	N	N
36	15	9	8.0	0.0000	0.0819	N	N	N	N
37	25	0	7.0	1.0000	0.9996	Y	Y	N	N
38	5	0	7.0	0.3333	0.6018	Y	Y	N	N
39	25	9	8.0	1.0000	0.7767	Y	Y	N	N
40	5	0	6.0	0.0000	0.6018	N	Y	Y	N
41	25	3	5.0	1.0000	0.9962	Y	Y	N	N
42	5	0	5.0	0.0000	0.6018	N	Y	Y	N
43	25	0	8.0	1.0000	0.9996	Y	Y	N	N
44	5	0	8.0	0.1667	0.6018	Y	Y	N	N
45	5	3	6.0	0.0000	0.1479	N	N	N	N
46	25	3	8.0	1.0000	0.9962	Y	Y	N	N
47	15	3	6.0	1.0000	0.8712	Y	Y	N	N
48	15	6	5.0	0.6667	0.4373	Y	Y	N	N

^1^ *P_o_*: observed adhesion ratio = number of wells showing adhesion/14; ^2^ *P_p_*: probability of adhesion calculated by Equation (7); ^3^ *P_o_* ≥ 0.1677: adhesion is considered to have occurred if observed adhesion ratio (*P_o_*) is ≥ 0.1677, designated as “Y”, otherwise “N”; ^4^ *P_p_* ≥ 0.1677: adhesion is considered to have occurred if calculated *P_p_* is ≥0.1677, designated as “Y”, otherwise “N”.

**Table 4:** Validation of logistic regression model and classification of biofilm formation and no-biofilm formation according to threshold (0.1667).

ID	T (°C)	NaCl (%)	pH	*P_o_* ^1^	*P_p_* ^2^	*P_o_* ≥ 0.1667 ^3^	*P_p_* ≥ 0.1667 ^4^	False Positive	False Negative
35	25	3	6.0	1.0000	0.9858	Y	Y	N	N
36	15	9	8.0	0.0000	0.0060	N	N	N	N
37	25	0	7.0	1.0000	0.9999	Y	Y	N	N
38	5	0	7.0	0.0000	0.0005	N	N	N	N
39	25	9	8.0	0.0000	0.9968	N	Y	Y	N
40	5	0	6.0	0.0000	0.0030	N	N	N	N
41	25	3	5.0	1.0000	0.7719	Y	Y	N	N
42	5	0	5.0	0.0000	0.0157	N	N	N	N
43	25	0	8.0	1.0000	1.0000	Y	Y	N	N
44	5	0	8.0	0.0000	0.0001	N	N	N	N
45	5	3	6.0	0.0000	0.0003	N	N	N	N
46	25	3	8.0	1.0000	1.0000	Y	Y	N	N
47	15	3	6.0	0.5000	0.1275	Y	N	N	Y
48	15	6	5.0	0.0000	0.0077	N	N	N	N

^1^ *P_o_*: observed biofilm formation ratio = number of wells showing biofilm formation/14; ^2^ *P_p_*: probability of biofilm formation calculated by Equation (8); ^3^ *Po* ≥ 0.1677: biofilm formation is considered to have occurred if observed biofilm formation ratio (*P_o_*) is ≥0.1677, designated as “Y”, otherwise “N”; ^4^ *P_p_* ≥ 0.1677: biofilm formation is considered to have occurred if calculated *P_p_* is ≥ 0.1677, designated as “Y”, otherwise “N”.

**Table 5:** Confusion matrices for validation of adhesion/no-adhesion or biofilm formation/no-biofilm formation of *L. monocytogenes* ST9, based on thresholds (0.1667).

	Adhesion/ No-Adhesion	Biofilm Formation/ No-Biofilm Formation
Accuracy rate	0.929	0.929
Precision rate	0.875	0.482
True positive rate (TPR)	1.000	0.929
True negative rate (TNR)	0.857	0.929
RMSE	0.285	0.291

## Data Availability

All data related to the research are presented in the article.
